# 

**Published:** 1882-05

**Authors:** 


### THE AMERICAN JOURNAL OF DENTAL SCIENCE,

EDITED BY

F. J. S. GORGAS, M. D., D. D. S.

S.G.O.L INDEXED

VOL. 16.—THIRD SERIES.

SURGEON GEN'LS OFFICE LIBRARY. 90212

BALTIMORE:
SNOWDEN & COWMAN.
LONDON:
TRUBNER & CO., 60 PATERNOSTER ROW.
1882 – 1883.

### *Pennsylvania State Dental Society.*

The fourteenth annual meeting of this Society will be held in Williamsport Pennsylvania, July 26th, at ten o'clock, A. M. Sessions continue three days.

Dentists having special cases available either in Operative, Mechanical or Surgical Clinics will please inform the Committee at once. Persons having new inventions or improvements of interest to the Profession will please notify the Committee, who will make arrangements to have them properly exhibited.

A full programme will be published hereafter.

G. W. KLUMP, *Ch'm of Com.*

WILLIAMSPORT, PA.

J. B. HODGKIN.

---

*Southern Dental Association.*—The approaching meeting of the Southern Dental Association, which convenes in Baltimore early in August next, should be borne in mind by all who are kindly disposed to that association. Full arrangements are being made to secure a large corps of clinical operators, culled from the ranks of the most gifted and experienced in the profession and covering not only Operative but Surgical and Mechanical Dentistry. It is proposed to make this *the distinguishing feature* of the meeting. The sessions of the Association will be held in the rooms of the Baltimore College of Dental Surgery, the ample Infirmary and Laboratory of which will furnish rare facilities for seeing as well as performing the operations &c., conducted. Further notice of the meeting and its details will be published hereafter.

J. B. HODGKIN.

### To Readers and Correspondents.

Communications intended for publication, and exchange journals and papers, should be addressed to the EDITOR OF THE AMERICAN JOURNAL OF DENTAL SCIENCE, 259 N. Eutaw St., Baltimore, Md. All letters relating to the business of the journal, or containing cash remittances, to be directed to Messrs. SNOWDEN & COWMAN. Articles for publication should be received by the editor at least three weeks previous to the first of the month on which the number in which it is designed for them to appear, is issued.

☞The American Journal of Dental Science will contain fifty pages of reading matter in each number, making a volume of about *Six Hundred* pages,

EXCLUSIVE OF ADVERTISEMENTS.

THE

### AMERICAN JOURNAL

OF

### DENTAL SCIENCE.

The *Journal* is issued on the first of each month and contains not less than *fifty* pages of reading matter in each number, exclusive of advertisements, making a volume of *six hundred* pages per annum.

In each number will be found Original Communications, Correspondence, Selected Articles, Monthly Summary, Bibliographical Notices and Editorial Matter, and every effort will be exerted to render the *Journal* worthy of the patronage of the Dental profession.

A generous co-operation is respectfully solicited from the members of the profession; and as the *Journal* has now a printing office of its own, which materially lessens the cost of its publication, it will be issued at the reduced subscription price of

**$2.50 Per Annum in Advance.**

Communications are respectfully solicited on all subjects pertaining to the practice of dentistry, as well as reports of cases occurring in dental practice.

### RATES OF ADVERTISING.

| | 1 MO. | 2 MO. | 3 MO. | 6 MO. | 12 MO. |
|---|---|---|---|---|---|
| 1 PAGE. | $15 00 | $22 50 | $30 00 | $50 00 | $100 00 |
| ½ " | 10 00 | 15 00 | 20 00 | 30 00 | 50 00 |
| ¼ " | 7 00 | 11 00 | 15 00 | 20 00 | 30 00 |
| ⅛ " | 5 00 | 8 00 | 11 00 | 15 00 | 20 00 |

All original communications designed for publication, works for review, new instruments for notice, exchanges, &c., should be directed to the editor, 259 N. Eutaw St., Baltimore, Md.

All advertisements and subscriptions should be addressed to

SNOWDEN & COWMAN,

*Publishers of the Am. Journal of Dental Science.*

86 W. FAYETTE ST. BET. CHARLES & LIBERTY STS.,

BALTIMORE, MD

